# Kinetic study, byproducts characterization and photodegradation pathway of profoxydim in a biochar water soil system

**DOI:** 10.1038/s41598-024-78621-x

**Published:** 2024-11-07

**Authors:** Álvaro Cervantes-Díaz, Juan Carlos Nieto-Carmona, Beatriz Sevilla-Morán, José Luis Alonso-Prados, Pilar Sandín-España

**Affiliations:** 1 Plant Protection Products Unit/Plant Protection Department, National Institute for Agricultural and Food Research and Technology INIA-CSIC, Ctra. La Coruña, Km. 7.5, Madrid, 28040 Spain; 2 Department of Agricultural Chemistry and Food Science, UAM-Madrid, Madrid, Spain

**Keywords:** Profoxydim herbicide, Photodegradation byproducts, HPLC-QTOF-MS/MS, Paddy water, Paddy soil, Biochar, Chemistry, Environmental chemistry, Environmental sciences, Environmental chemistry

## Abstract

The study focused on the photodegradation of profoxydim, a low-toxicity cyclohexanedione herbicide commonly used in rice crops, under simulated sunlight conditions. Profoxydim’s behavior in paddy field conditions is not well understood, and this research aimed to fill that gap, particularly examining the effect of commonly utilized organic amendments such as biochar (BC) on its degradation. Results indicated that profoxydim degrades rapidly, with a half-life of 2.4 ± 0.3 h in paddy water and 1.03 ± 0.1 h in paddy soil. However, when BC was introduced, the degradation slowed significantly, extending the half-lives to 3.1 ± 0.2 h in water and 3.07 ± 0.5 h in soil. The study identified five degradation products (DPs) using TOF mass accuracy measurements and MS/MS spectra fragmentation. Two of these DPs were found to be more stable than profoxydim itself. Additionally, the research proposed a novel photodegradation pathway, highlighting processes such as homolytic C-N bond cleavage, photoisomerization, and photoinduced oxidation. The study’s findings contribute new insights into the environmental fate of profoxydim, offering a deeper understanding of its transformation in rice paddy fields and aiding in the assessment of potential risks associated with its residues in agricultural environments.

**Supplementary Information:**

The online version contains supplementary material available at 10.1038/s41598-024-78621-x.

## Introduction

Weeds are considered the greatest challenge affecting rice production in Europe, and ineffective control of weeds such as *Echinochloa* spp. and *Cyperus* spp. can result in a severe reduction in crop yields^1^. Thus, the use of herbicides, such as the cyclohexanodione profoxydim, has become a fundamental tool in the management of rice fields, and it is probable that this will become more significant in the future due to the impact of climate change^2^. The presence of herbicide residues in paddy fields is a problem of environmental concern since the herbicides occurring in floodwater can contaminate surface water bodies connected with rice fields^3^. Previous works have reported the presence of imidazolinone herbicides in the outlet water of rice fields and in surface waters^4^. The persistence of pesticides is partly linked to the different degradation pathways that these compounds can undergo in the environment, such as microbial degradation, chemical hydrolysis and photochemical reactions. Profoxydim is one of a limited number of herbicides approved for use in rice cultivation and belongs to the last-generation cyclohexanedione oxime family. It is widely used for the postemergence control of grassy rice weeds such as *Echinochloa crusgalli* and acts as an inhibitor of acetyl coenzyme A carboxylase activity in chloroplasts. A limited number of studies have investigated its persistence in the paddy environment. Sanchez et al.^5^ and Tsochatzis et al.^6^ observed a rapid dissipation of profoxydim in paddy soil below one day, indicating that it is nonpersistent in paddy fields. In our previous work, the biological degradation of profoxydim was studied in different soils under laboratory conditions^7^, and the results showed that the degradation of profoxydim was governed mainly by microbial processes with half-lives of 0.53 days.

Rice cultivation thrives in flooded conditions, requiring careful management of water levels. Paddy fields are intentionally flooded to a depth of 5–10 cm, with soil continuously soaked. Before harvest, fields are drained to allow the soil to dry. In light of the fact that photolysis is acknowledged to be the primary degradation process that takes place in natural water systems^8^, there is a clear need for in-depth investigation into photodegradation phenomena in paddy soil and paddy water. However, the photodegradation of profoxydim in paddy water or paddy soil is currently unknown. It is therefore important to determine the photochemical behaviour and assess the risk of profoxydim under paddy field conditions. Cyclohexanedione oxime herbicides are highly photosensitive and easily degraded in natural waters, and direct photolysis is their main degradation route in aquatic environments, with a half-life of a few hours in irradiated waters^9^. In addition, the presence of natural water constituents, such as ions or dissolved organic matter, has different effects on the photodegradation rates of herbicides^10^.

Currently, soil amendment strategies such as the addition of biochar (BC) are being developed to improve paddy soil quality and increase crop productivity^11–14^. Moreover, interest has increased in BC application to soils not only for its benefits as an organic fertilizer but also due to the need to address climate change^11,15,16^. However, BC amendments alter the physicochemical properties of soils and influence soil processes, such as water- and nutrient-holding capacities^17^. Since BC application alters soil and water chemistry, it could have effects on the environmental behavior of herbicides^18^. However, studies on the effect of the addition of BC on the photodegradation of pesticides are limited, and few studies have addressed this issue^19^. Zeng et al.^13^ reported an increase in the amount of photogenerated reactive intermediates, such as hydroxyl radicals, in BC-amended paddy water. The aim of this work is to elucidate to what extent BC affects the photodegradation of profoxydim.

Importantly, most studies on herbicides are mainly focused on the fate and toxicity of the active compounds rather than on the identification of the metabolites derived from the active compound and their effects. Many studies have confirmed that DPs are easily transported into natural waters. This can lead to water quality degradation. In fact, DPs can be more persistent, toxic and/or mobile, posing greater risks than their parent compounds^20–22^. As such, attention should be directed toward the effects of DPs in addition to those of the parent compound. The identification of DPs and evaluations of their biological activities and toxicological properties are of utmost importance since these might be ‘relevant for overall approval decisions or for defining risk mitigation measures’^23^. The DPs of the cyclohexanedione herbicides clethodim and alloxydim^24^ showed greater ecotoxicity and a greater risk of contaminating natural waters than did the corresponding active substances. However, there is a lack of evidence concerning the degradation products (DPs) and degradation pathway of the photodegradation of profoxydim.

The main objective of the present work was to address the lack of information on the photodegradation pathways and byproducts of profoxydim in the water and soil of a paddy field and the effect of BC addition. In this work, an exhaustive and detailed MS/MS fragmentation study and accurate mass measurements were performed to elucidate the structure of each generated photoproduct. Furthermore, for the first time, a complete photodegradation reaction mechanism was proposed for profoxydim.

## Materials and methods

### Chemicals and paddy water and soil samples

The profoxydim analytical standard [(*E*)-2-{1-[2-(4-chlorophenoxy)propoxyimino]butyl}-3-hydroxy-5thian-3-yl-cyclohex-2-enone] (98.7% purity) was purchased from HPC Standards GmbH (Cunnersdorf, Deutschland). Acetonitrile (HPLC superGRAD grade) and methanol (HPLC grade) were acquired from Macron Fine Chemicals (Pennsylvania, USA), and formic acid was obtained from Scharlab S.L. (Barcelona, Spain). The ultrapure water used for the LC mobile phase and the aqueous solutions were obtained from a Millipore system, 18 mΩ, Milli-Q-50 (Millipore, Milford, MA, USA). Filters (regenerated cellulose, 0.45 μm) were obtained from Sartorius Biotech (Gotinga, Germany).

The soil and water samples used in this study were collected from a paddy field in Seville (37°12′27.4′′ N 5°49′13.1′′ W), one of the most important rice-growing areas of Spain. The water samples were collected in 1 L glass bottles (Duran, Mainz, Germany), vacuum-filtered (regenerated cellulose filters, 0.45 μm pore size, Sartorius Biotech) and kept refrigerated at 4 °C until irradiation. The soil samples were collected from the 0–10 cm layer, air-dried at room temperature, crushed to pass through a 2 mm sieve and kept in a cool and dark environment before use. Samples of soil and water from the field amended with BC were collected in the same way as previously described. The physicochemical properties of the paddy soil and paddy water are described in Table S1.

### Photodegradation experiments

Photodegradation experiments were performed using a Suntest CPS + sunlight simulator from Atlas (Linsengericht, Germany) equipped with a xenon arc lamp (1500 W) and a special UV glass filter which limits the transmission of wavelengths below 290 nm (experimental device is shown in Fig. S1). Photochemical studies were performed at an irradiation intensity of 500 W/m^2^, and the temperature was maintained at 25 ± 1 °C using an AtlasSunCool chiller unit. The sunlight simulator provided a spectral distribution close to that of natural sunlight and constant irradiance that allowed for experiments to be performed under reproducible irradiation conditions, avoiding variations caused by geographical, seasonal or climatic conditions.

Profoxydim solutions (4 mg/L) were prepared in ultrapure water, paddy water and BC-amended paddy water. A profoxydim control sample wrapped in aluminum foil was also placed in the sunlight simulator for testing for profoxydim hydrolysis. Twenty milliliters of each solution was exposed to simulated solar radiation in capped cylindrical quartz cuvettes with magnetic stirring. Aliquots of 1 mL were withdrawn at given time intervals and immediately analyzed to determine reaction kinetics via HPLC-DAD.

To perform the photodegradation of profoxydim in paddy soil, 4 g each of unamended paddy soil and BC-amended paddy soil were weighed in Petri dishes (Ø = 4 cm). A solution of 2 mL of profoxydim (10 mg/L) was pipetted onto the Petri dishes, corresponding to the field dose. The dishes were exposed to simulated solar light.

To analyze the dissipation of profoxydim in paddy soil samples, extraction was performed with the QuEChERS method^25^. This method was previously developed by our group^7^, the extraction efficiency of profoxydim in soil was 82.5 ± 2.4%. Briefly, 8 mL of solution (40% acetonitrile:60% methanol) was added to the soils in 50 mL polypropylene centrifuge tubes and shaken for 1 min. To extract profoxydim, citrate-buffered EN (4.0 g anhydrous MgSO_4_, 1.0 g NaCl, 0.5 g sodium citrate dibasic sesquihydrate and 1.0 g sodium citrate tribasic sesquihydrate) (HPC Standards GmbH (Cunnersdorf, Germany) was added, and the mixture was shaken for 1 min. The samples were centrifuged at 3000 rpm at 4 °C for 5 min, and the supernatant was analyzed by HPLC-DAD.

All kinetic experiments were performed until the herbicide was completely degraded. Three replicates were carried out for each water and soil photodegradation experiment, and the numerical results presented correspond to the mathematical average of these three independent analyses.

### Analytical methods

For the kinetics studies, the profoxydim concentrations were determined via HPLC-DAD (Agilent Technologies 1100 series model; Agilent Technologies, Palo Alto, US). The mobile phase was a mixture of ultrapure water with 0.1% formic acid (A) and acetonitrile (B). The flow rate was 1 mL/min, the injection volume was 20 µL, and an isocratic method based on 90% acetonitrile was used. Separations were performed on a Waters C18 Atlantis column (3 μm particle size, 4.6 mm × 150 mm) (Waters, Dublin, Ireland). The profoxydim retention time under these conditions was 6.2 min.

The identification and analysis of the evolution of the DPs of profoxydim were carried out using an HPLC system (Series 1100; Agilent Technologies, Palo Alto, USA) coupled to a hybrid QTOF mass spectrometer (QStar Pulsar I, Applied Biosystems) equipped with a Waters C18 Atlantis column (3 μm particle size, 4.6 mm × 150 mm). A gradient method was developed to separate and detect as many DPs as possible. Mobile phases A and B were ultrapure water with 0.1% formic acid and acetonitrile, respectively. The proportions of (B) in the mobile phase were as follows: 0–4 min, 23%; 4–17 min, 23–62%; and 17–20 min, 62–90%. The flow rate was 0.7 mL/min, and the injection volume was 20 µL. The analyses were performed in positive and negative ion modes. The instrumental parameters were a mass range of 50–1200, an ion spray voltage (IS) of 5500 V, an ion source gas pressure (GS1) of 50 psi, an ion source gas pressure (GS2) of 55 psi, a curtain gas pressure (Cur) of 20 psi, a declustering potential (DP) of 70 V, a focusing potential (FP) of 210 V, and a declustering potential of 2 of 15 V. The elemental composition calculator in the Analyst software (Applied Biosystems) was used to process the precise masses obtained.

### Statistical analysis

The degradation of profoxydim in the irradiated media was postulated to follow first-order kinetics (SFO) given by the following equation:

1 $${C_t}={C_0}{e^{ - kt}}$$

where C_0_ and C_t_ are the concentrations of the herbicide at time 0, t is the irradiation time (min), and k is the rate constant (/min) of the photolytic process.

The rate constants of photolysis were determined from the plots of herbicide concentrations vs. irradiation time using a nonlinear regression fit. The half-lives of photolysis for profoxydim (t_1/2_), the time required to reduce the initial herbicide concentration by 50%, were determined from the rate constants previously calculated by the following formula:

$${t_{{\raise0.7ex\hbox{$1$} \!\mathord{\left/ {\vphantom {1 2}}\right.\kern-0pt}\!\lower0.7ex\hbox{$2$}}}}={\raise0.7ex\hbox{${\ln 2}$} \!\mathord{\left/ {\vphantom {{\ln 2} k}}\right.\kern-0pt}\!\lower0.7ex\hbox{$k$}}$$

For the experiments that did not yield a good fit to first-order kinetics, a biphasic kinetic model^26^, the Gustafson and Holden model (FOMC), was postulated. The equation to determine the kinetic parameters is given by the following equation:

2 $${C_t}={\raise0.7ex\hbox{${{C_0}}$} \!\mathord{\left/ {\vphantom {{{C_0}} {{{\left[ {\left( {t/\beta } \right)+1} \right]}^\alpha }}}}\right.\kern-0pt}\!\lower0.7ex\hbox{${{{\left[ {\left( {t/\beta } \right)+1} \right]}^\alpha }}$}}$$

In this case, the half-life of profoxydim was determined via the following equation:

$${t_{{\raise0.7ex\hbox{$1$} \!\mathord{\left/ {\vphantom {1 2}}\right.\kern-0pt}\!\lower0.7ex\hbox{$2$}}}}={\beta}\left[ {{2^{\left( {\frac{1}{\alpha }} \right)}} - 1} \right]$$

A statistically significant difference between the half-lives of profoxydim under the different experimental conditions studied was determined using one-way analyses of variance (ANOVA) at the 0.05 significance level.

## Results and discussion

### Photodegradation kinetics in paddy water

Photodegradation studies were performed in a Suntest reactor that provides a wavelength distribution close to that of natural sunlight and constant irradiance, thereby minimizing environmental variability. The kinetic parameters obtained with this device show good correlations with those under natural sunlight and those obtained with a filtered xenon arc lamp^24^.

Despite the fact that few herbicides absorb solar radiation (λ > 290 nm) or they do so at low efficiencies, the UV absorption spectrum of profoxydim overlaps with solar emission at 300–350 nm (Fig. S2), which means that the process of direct photolysis is feasible. According to the European Commission, 2009^23^, phototransformation must be considered if the molar extinction coefficient of a compound is > 10 L/mol cm at λ ≥ 290 nm. The ε value obtained at λ = 290 nm in aqueous solution at natural pH for profoxydim was 16,618 L/mol cm, which indicates that direct photolysis can contribute significantly to its degradation in natural waters and should be taken into account when studying its fate in the environment.

**Fig. 1:**
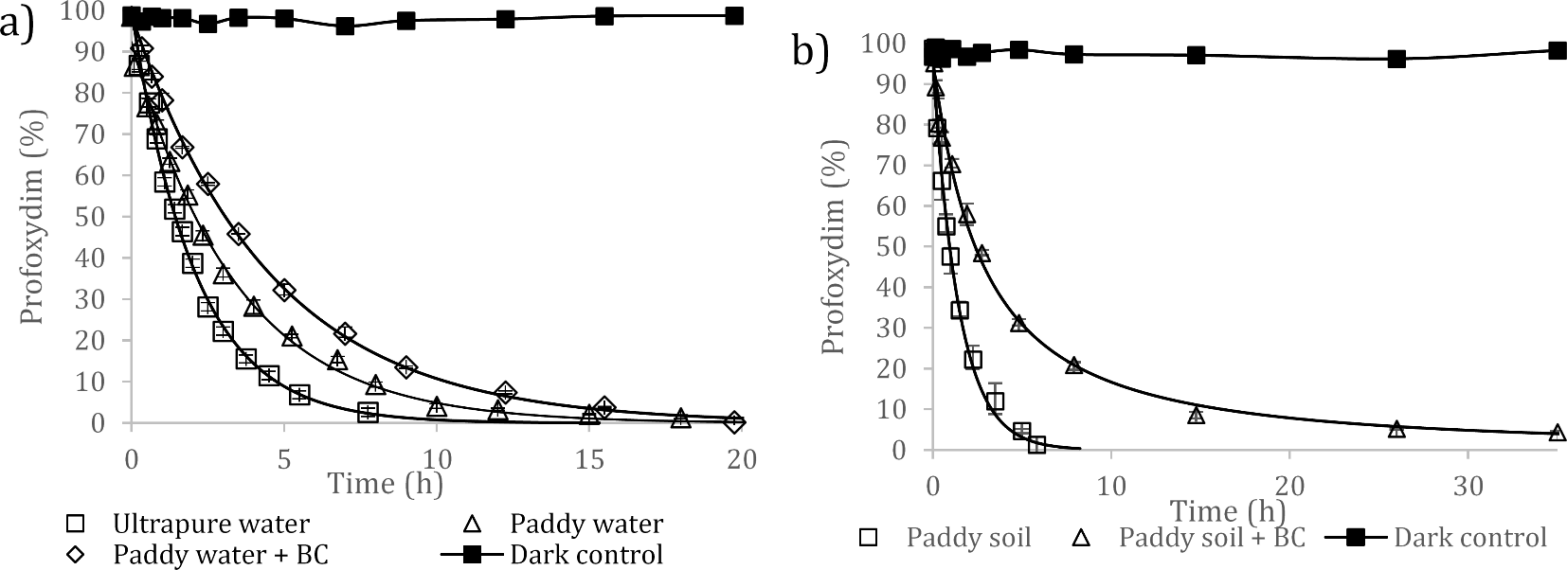
Photodegradation kinetics of profoxydim in the paddy system: (a) water and (b) soil. The results are the average of three replicates.

The photodegradation kinetics of profoxydim were fitted to the single first-order (SFO) model, and the results are shown in Table 1. The experimental data fit the SFO model well, yielding R^2^ values greater than 0.99 (Fig. 1a). Additionally, during the irradiation experiments, control samples in the absence of radiation were tested to rule out other possible degradation processes. The concentration of the herbicide remained constant during the entire exposure period, allowing us to eliminate reactions that were not photoinitiated, such as thermolysis or hydrolysis. As shown in Fig. 1a, profoxydim degrades quite quickly in water and is completely degraded after 10 h in ultrapure water, with a half-life of 1.4 ± 0.2 h. In paddy water, degradation is slower, with a half-life of 2.4 ± 0.3 h, and 15 h is required for the complete degradation of the active substance.

**Table 1:** Kinetic parameters (mean ± standard deviation) for the degradation of profoxydim in different matrices (paddy water and paddy soil).

Matrix	Kinetic model	Degradation constant	t_1/2_ (hours)	*R* ^2^
Ultrapure water	SFO	k = (0.483 ± 0.009) h^− 1^	1.4 ± 0.2a	0.998
Paddy water	SFO	k = (0.293 ± 0.010) h^− 1^	2.4 ± 0.3b	0.995
Paddy water + BC	SFO	k = (0.221 ± 0.005) h^− 1^	3.1 ± 0.2c	0.998
Paddy soil	SFO	k = (0.67 ± 0.04) h^− 1^	1.03 ± 0.1a	0.995
Paddy soil + BC	FOMC	α = 1723.04 ± 57.4 β = 7640.7 ± 217.3	3.07 ± 0.5b	0.991

Single first-order kinetic model (SFO) and first-order multicompartment model (FOMC). Different letters indicate significant differences according to the least significant difference test (LSD) at a significance level of 95%.

Zeng et al.^13^ revealed that due to flooding conditions and fertilization, paddy water contains high levels of DOM, which changes the water chemistry. In our case, water analysis (Table S1) revealed a very high organic matter content (71.0 mg/L) in paddy water compare to ultrapure water (1.5 mg/L). However, in the literature, the presence of DOM has been shown to have different effects on the photodegradation of pesticides. On the one hand, the excited states of DOM can participate in charge-transfer interactions with compounds or generate reactive intermediates, such as hydroxyl radicals, singlet oxygen or hydrogen peroxide^27^, that act as “sensitizers” and accelerate the degradation of the compounds. Conversely, DOM can act as an “optical filter”, trapping photons or scattering incident radiation^28–30^, thereby decreasing the degradation rate. In our study, the presence of organic matter reduced the degradation of the herbicide by a factor of two, suggesting that DOM substances can absorb some of the photons emitted by sunlight, consequently slowing down direct photochemical reaction processes. These results are consistent with those of previous studies conducted by our research group, in which the presence of varying concentrations of one of the primary organic matter components in natural waters, specifically humic acid (1–20 mg/L), was found to retard the degradation of other cyclohexanodione herbicides. The half-lives for clethodim ranged from 92 to 196 min and for alloxydim, from 45 to 104 min^10^. The findings demonstrate that cyclohexanedione herbicides undergo rapid degradation, rendering them non-persistent compounds in aqueous solutions under solar irradiation. Consequently, the probability of water contamination by cyclohexanedione herbicides is minimal.

Organic amendments are widely used for the improvement of crop and soil properties. However, soil amendments can alter the concentration and structure of DOM in paddy water. Zeng et al.^13^ observed that the application of BC resulted in an increase in DOC in paddy water because amendments decomposed to form DOM, mainly carboxyl-rich acyclic molecules and unsaturated hydrocarbons. The photodegradation of profoxydim in paddy water + BC was well fitted to a first-order kinetic model. The half-life increased to 3.1 ± 0.2 h. In previous studies conducted by our research group, we observed that the photodegradation of other cyclohexanedione herbicides was also well-represented by a first-order kinetic model. In these studies, the addition of organic matter to natural waters or the introduction of humic acids resulted in a reduction in the photolysis rate. The half-life of alloxydim increased from 139.04 to 203.08 min, while that of clethodim increased from 28.09 to 147.0 min with the addition of humic acids^8^. The results of the ANOVA analysis indicated that there were significant differences in photodegradation between the three types of water studied (ultrapure, paddy and BC-amended paddy water) (Table 1). In conclusion, the addition of BC amendments to DOM in paddy water significantly inhibited the photodegradation of profoxydim in comparison to the degradation observed in ultrapure water. The results demonstrate that the primary factor influencing the degradation of profoxydim in water is direct photolysis, with indirect photolysis also occurring to a lesser extent.

Few publications exist in literature referring on the role of these carbon materials in the photodegradation of organic contaminants^31–35^, especially of pesticides in aquatic and soil environments.

Biochars due to their high specific surface area, microporosity and aromaticity, as well as the presence of O-containing functional groups such as carboxyl, hydroxyl, and phenolic surface functional groups may act as effective sorbents for organic pollutants in soil and water. Furthermore, due to its amorphous structure, biochar is capable of incorporating a range of different adsorption mechanisms, including pore filling, electrostatic interaction, the hydrophobic effect of hydrogen bonding, and π-π interaction^31,32^. With the wide application of biochar, increasing amounts of DOM are released to the environment. In our study the soil DOM increased from 1.26 to 6.49% with biochar. In paddy water the DOM increased from 71.0 to 102.4 mg/L.

Regarding aqueous environments, the presence of biochar can exert different effects as the organic matter dissolved is highly increased. On one hand DOM is an important photosensitizer by generating reactive oxygen species (ROS) such as singlet oxygen, superoxide anion free radical, hydrogen peroxide and hydroxyl radicals than can enhance the degradation of pollutants^36,37^ while on the other hand increasing concentration of biochar DOM absorb a significant fraction of the simulated solar light, acting as an optical filter, reducing the exposure of the pollutant molecules to the radiation and thus reducing the direct photolysis of the compound. Serelis et al.^34^ observed that the photodegradadation of the herbicide metribuzin gradual decrease as the BC concentration increase. In addition, higher concentrations of biochar particles and DOM may inhibit the removal of the compound through the interactions between DOMs and the analyte^33,38^). Nevertheless, the inhibition dynamics of binding to their photodegradation of DOM/organic complexes are still unclear. Lower photolysis rates were previously observed in DOM rich waters for metribuzin or other triazinic herbicides, like atrazine^39,40^ or imidacloprid^35^. In our study, the presence of a high concentration of biochar slow down the degradation rates of profoxydim in water environments. Further studies are needed to go deeper into the mechanism behind the retarding effect of BC-DOM.

### Photodegradation kinetics in paddy soil

In soils, photolysis occurs within a shallow surface zone, the depth of which depends on soil characteristics and the mechanism of photodegradation^19^. Direct photolysis on the soil surface is limited to a region of approximately 0.2–0.3 mm. Moreover, light absorption and photolysis are influenced by sorption reactions that are related to soil organic matter content^41^. Figure 1b shows the photodegradation of profoxydim in paddy soil under simulated sunlight. The data were fitted to a first-kinetics order, resulting in a half-life of 1.03 ± 0.1 h for the paddy soil samples (Table 1). In a previous work, the biodegradation of profoxydim in paddy soil in the absence of light resulted in a profoxydim half-life of 1.48 days^7^, which was slightly greater than the half-life observed under photodegradation in this study (1.03 h). This suggests that photolysis is also one of the main degradation routes for this herbicide in the environment. Few studies have investigated the degradation of profoxydim in paddy field conditions and none of them in the presence of organic amendments. Sanchez et al.^5^ and Tsochatzis et al.^6^ conducted paddy field dissipation studies and obtained half-lives of less than one day, indicating that the active substance does not pose a risk to adjacent natural water resources. Nevertheless, it is challenging to make a direct comparison between the degradation behaviour of profoxydim as reported in different studies due to the influence of different field conditions, which introduce a degree of variability that makes a direct comparison difficult^5,6^.

With respect to paddy soil, we aimed to investigate the effect of BC addition to paddy soil on profoxydim photodegradation. The application of BC to soil significantly modified the degradation kinetics of profoxydim relative to unamended soil (Table 1). ANOVA revealed that photodegradation in BC paddy soil significantly increased the half-life from 1.03 ± 0.1 h (paddy soil) to 3.07 ± 0.5 h. Even after 35 h, complete degradation did not occur. The degradation kinetics were fitted to a biexponential model (FOMC), which consisted of a relatively fast initial phase followed by a slower phase. This behavior could be explained by two competing processes, photodegradation, which contributes more to the first step, and absorption, which is predominant in the second step. In the presence of BC significant differences were found between the water samples (p-value: 0.000) and between the soil samples (p-value: 0.000), showing in both cases the addition of biochar resulted in higher values of degradation rates. These ANOVA’s results are shown in Figs. S3 and S4.

Haskis et al.^42^ in line with our results, employed a biexponential model (FOMC) to investigate the photodegradation of metribuzin in soil with the addition of BC. In a prior investigation the biodegradation of profoxydim, the application of organic amendments was observed to significantly enhance the sorption of profoxydim. This was attributed to an increase in soil organic carbon, which led to a reduction in the biodegradation rate and an increase in the persistence of the herbicide within the soil^7^.

In conclusion, the photodegradation rates of profoxydim exhibited no significant difference between the paddy water and paddy soil matrices. However, a decrease in degradation was observed in the presence of BC in both matrices. These results underline the importance of considering this abiotic process when studying the environmental behavior of this herbicide and the need to study the resulting byproducts generated in the media.

The current work was performed at the laboratory scale under simulated sunlight to assess the fate of profoxydim under specific paddy conditions. More research is necessary to confirm the results of this work under different agronomic and climatic field conditions.

### MS/MS fragmentation study and identification of DPs

**Fig. 2:**
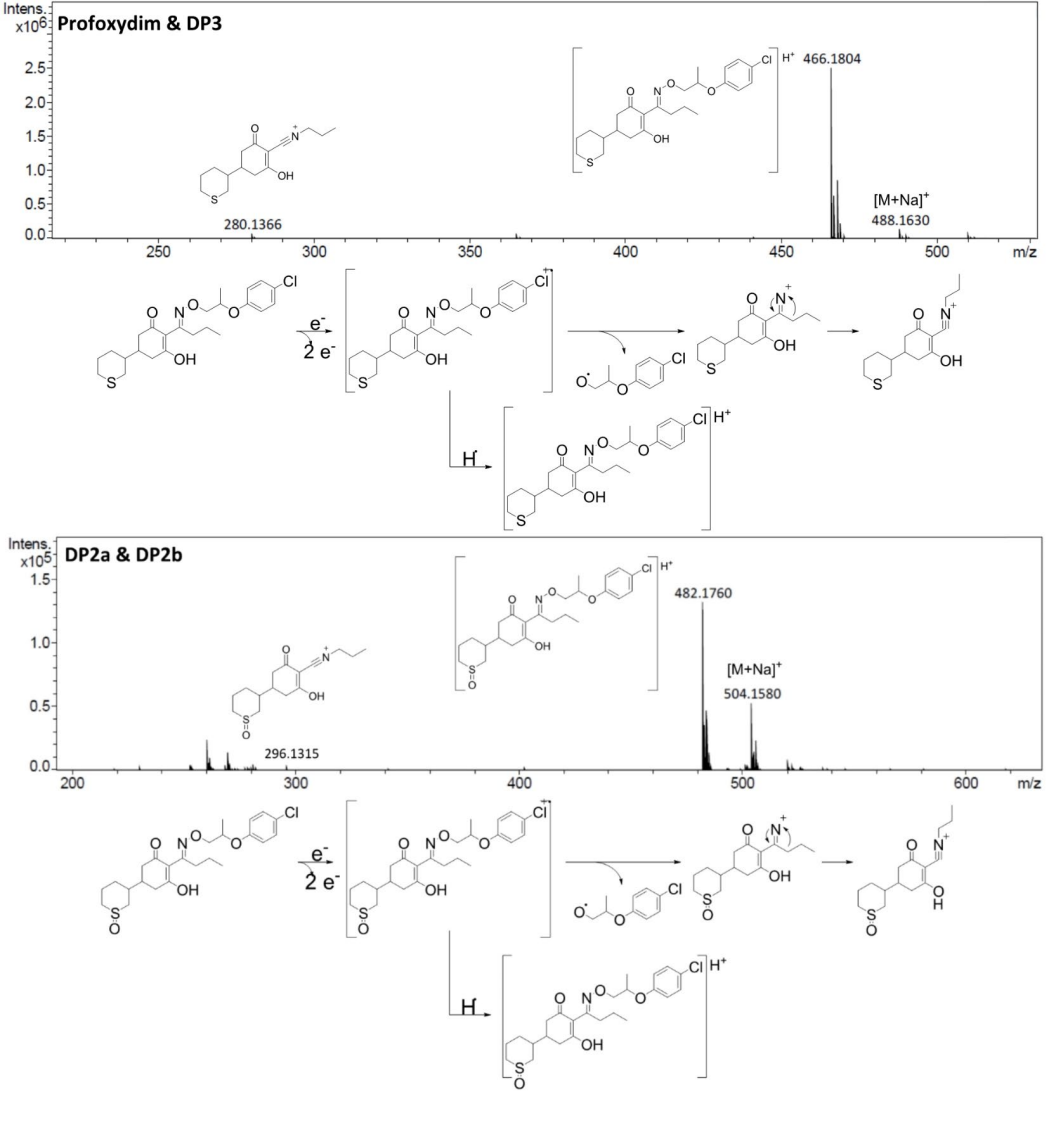

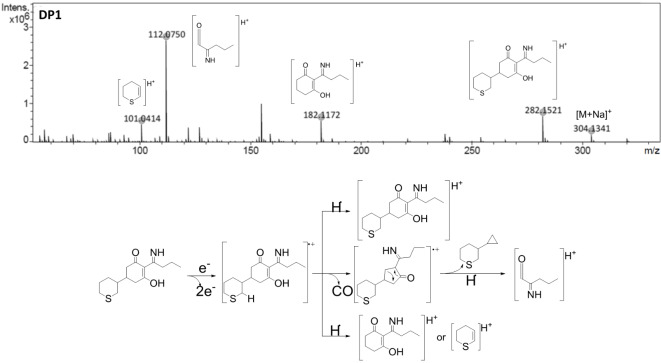
LC-QTOF mass spectra and fragmentation pathway of profoxydim and its major DPs (DP1, DP2 and DP3).

To elucidate the structure of the generated DPs, HPLC-QTOF-MS/MS analysis was performed, a commonly used techniques for the identification of DPs^29,30,43^. The interface employed (ESI) allowed us to obtain good fragmentation of each photodegradation product detected. A better fragmentation response was obtained in the positive mode than in the negative mode. Thus, the MS/MS fragmentation pattern of each compound helped us to elucidate their structures. To identify the DPs, various samples from water, soil and BC-amended soil and water experiments were analyzed. We detected five DPs, and we observed the same compounds in all the matrices with similar evolution profiles. Figure 2 shows the MS/MS spectrum of each compound and its respective fragmentation pathways. The elemental composition of all the product ions was confirmed by accurate mass measurements. MS/MS fragmentation of the profoxydim group was studied to facilitate subsequent interpretation of the MS/MS spectra of the unknown byproducts. The fragmentation pathway of the profoxydim group is based on the cleavage of oxime, which produces ammonium.

Six DPs of profoxydim were identified previously by our group in the biological degradation of profoxydim in soil^7^. Under photodegradation conditions, the same compounds were identified. The main difference is that the majority of DPs are profoxydim-imines. In contrast, for biological degradation, the main DP was a profoxydim-sulfoxide generated by the oxidation of sulfur atoms. Nevertheless, in this study, a comprehensive and thorough examination of fragmentations and their associated mechanisms was conducted, representing a novel contribution to the existing literature.

The signal for the profoxydim group at m/z 466.1804 corresponds to that of the protonated molecule [M + H]^+^. The product ion at m/z 280.1366 was produced by the loss of the oxime moiety to form an imine group. The loss of oxime produced a decrease of 186 Da due to the loss of [C_9_H_11_O_2_Cl].

**Fig. 3:**
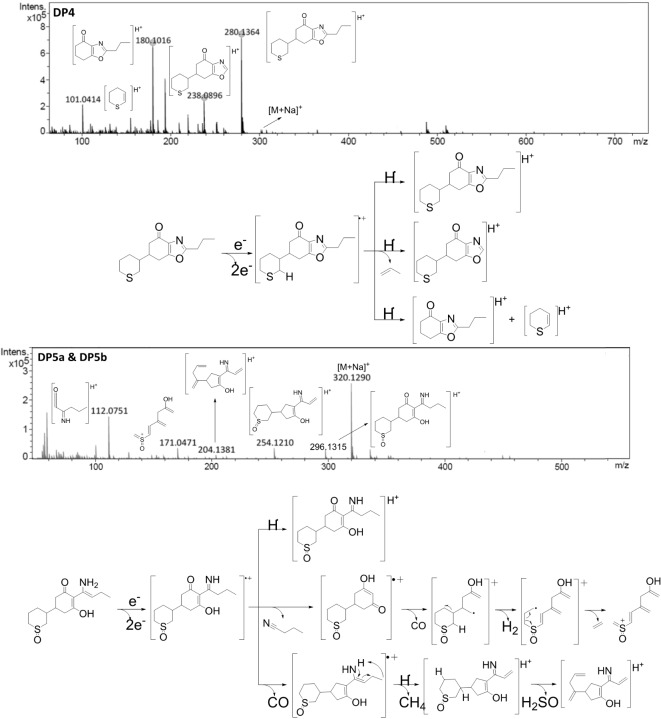
Photodegradation of profoxydim in paddy water. (a) LC-DAD chromatogram of profoxydim at the beginning of photodegradation and LC-DAD chromatogram of profoxydim at the end of the irradiation. (b) Evolution of degradation products (empty symbols and dotted lines) formed during the photolysis of profoxydim (filled symbols and solid line).

The photodegradation product DP3 shows an MS/MS fragmentation pattern identical to that of profoxydim. Therefore, it corresponds to the Z-isomer of profoxydim. Several authors have reported that the *E*-isomer of cyclohexanedione herbicides may equilibrate with the *Z*-isomer under different conditions^44,45^. The E-isomer is predominant because it facilitates the formation of hydrogen bonds between the oxime and enol groups. Profoxydim appeared at 52.8 min in the chromatogram (Fig. 3A), whereas DP3 appeared at 44.2 min, indicating an increase in polar character. This increase in polarity can be explained by the fact that the *Z*-isomer cannot form intramolecular hydrogen bonds and exposes more polar groups to the stationary phase of the column. Previous studies performed by our group have shown that the formation of the *Z*-isomer of cyclohexanedione herbicides occurs under simulated radiation^9^. Furthermore, this DP has also been detected in the biological degradation of profoxydim in paddy soil^7^.

The main byproduct (DP1) presented a signal at m/z 282.1521, which corresponded to the protonation of the profoxydim-imine formed due to the transformation of the oxime group. The MS/MS spectra of DP1 revealed three major fragments (Fig. 2). The first fragmentation could be attributed to the cleavage of the C-C bond between two rings, which produced fragments at 182.1172 and 101.0414. Only one fragment could be formed per molecule, depending on where the radical cation was produced during ionization; the other fragment would not be detected because it would be a neutral product.

The major fragment at m/z 112.0750 corresponds to the elemental composition C_6_H_10_NO^+^, which could result from a two-stage fragmentation. First, there would be a loss of carbon monoxide, forming a cyclopentanone, which would lead to the formation of a ketene cation and the loss of a bicyclic neutral compound^46^. The main byproduct of the photodegradation of cyclohexanedione herbicides forms via the photolysis of the N-O bond of the oxime to yield the corresponding imine^24^.

**Fig. 4:**
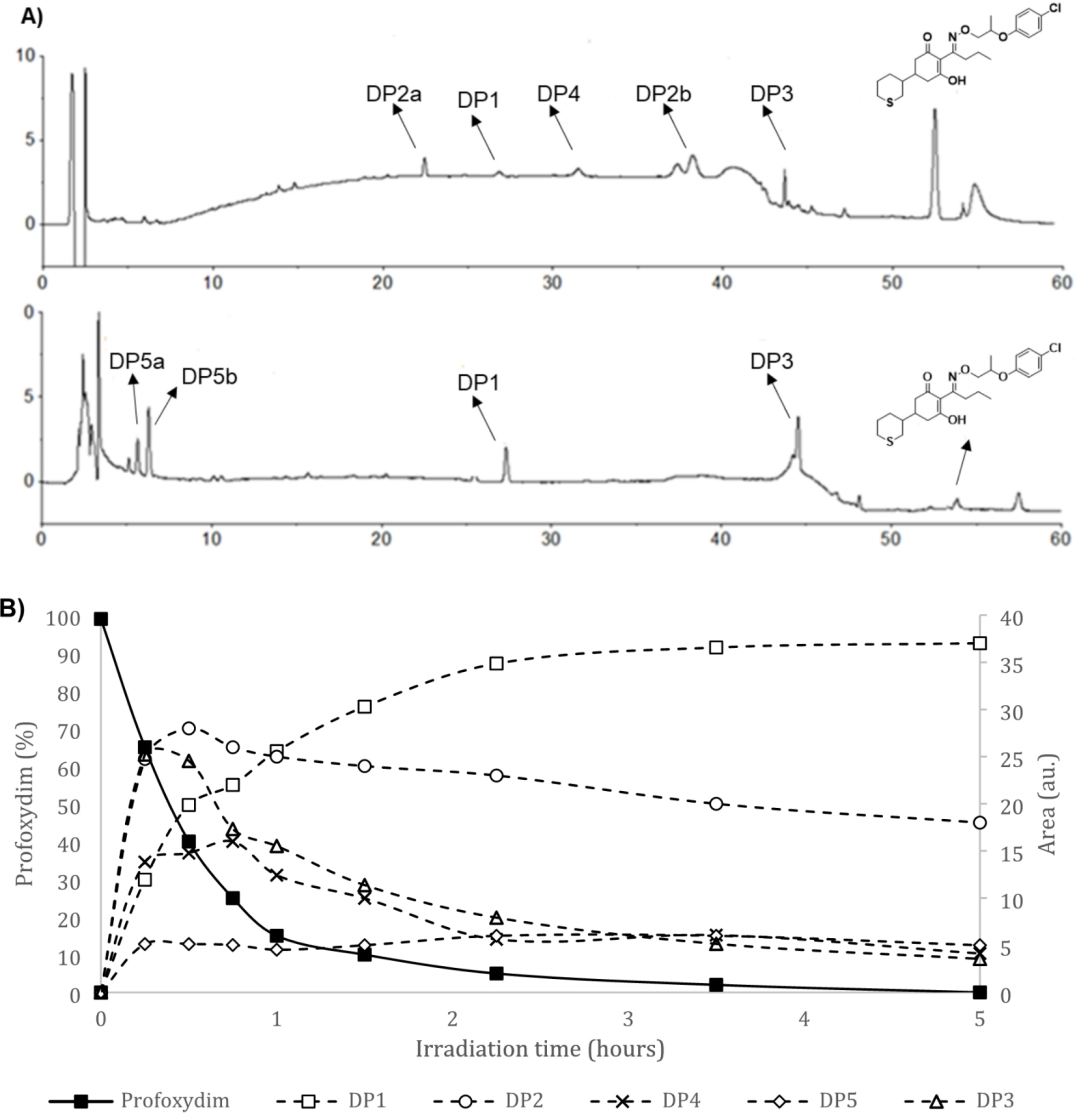
LC-QTOF mass spectra and fragmentation pathway of minor byproducts (DP4 and DP5).

The nominal masses of DP2a and DP2b appeared at m/z 482.1762, which is 16 Da greater than that of profoxydim, indicating the presence of one additional oxygen atom. The MS/MS spectra (Fig. 4) presented a chlorine and sulfur isotopic signature, showing the presence of the oxime and sulfur chain, respectively, which corresponded to the sulfoxidation reaction that produces profoxydim-sulfoxide. The oxidation of sulfides to sulfoxides occurs readily in this kind of compound^47^, and it generates a new asymmetric center in the compound, which is expected to result in different peaks due to the separation of the diastereoisomers (DP2a and DP2b). The spectra of DP2 (Fig. 3) presented a fragment at m/z 296.1315, corresponding to the loss of the oxime moiety (186 Da; [C_9_H_11_O_2_Cl]) in the same way as that observed for the profoxydim and its isomer (DP3). DP2 presented the same mechanism of fragmentation as profoxydim because the only difference between the two molecules is the sulfoxide group. DP2 presented the same structure as profoxydim with a sulfoxide group; therefore, the mechanism of fragmentation was the same.

The MS/MS spectrum of DP4 (Fig. 4) showed a signal at m/z 280.1364, which corresponds to the protonated molecule [M + H]^+^ with an elemental composition of C_15_H_21_NO_2_S. This compound came from the loss of 188 Da in profoxydim, which might have corresponded to the loss of the remaining -OR group in the oxime ([C_9_H_11_O_2_Cl]) and to the loss of a hydrogen molecule. The oxazole formed from the oxime has this elemental composition, which is why it was considered the most likely structure. Different authors have shown that the formation of oxazole is typical in the photodegradation of cyclohexanedione herbicides^48,49^. DP4 presented three important fragments: the first fragmentation occurred via inductive cleavage assisted by β-hydrogen removal from the alkyl chain of the oxazole, with a signal at m/z 238.0896, and the release of propene as a neutral compound. For the formation of the other two fragments, something similar to that described for the imine DP1 occurred (Fig. 2), in which the breakage of the bond between the two rings led to the formation of two molecules: the bicyclic compound or the dihydrothiopyran. One of these molecules would be neutral and undetectable, and the other would form a cation during the initial ionization, producing two signals at m/z 180.1016 and 101.0414.

The nominal masses of DP5a and DP5b were m/z 298.1471 (Fig. 4). This molecule corresponded to sulfoxidation and the loss of the oxime moiety, which produced the imine of profoxydim-sulfoxide. A new chiral center was generated during sulfoxidation; therefore, two isomers were present. DP5 presented two different fragmentation pathways. In the first case, chain fragmentation started with the rupture of a cyano residue, followed by the loss of carbon monoxide and finally the formation of the [C_8_H_11_O_2_S]^+^ fragment at m/z 171.0471. In the second pathway, the loss of carbon monoxide, methane (inductive cleavage assisted by β-hydrogen removal) and sulfinyl hydride led to the formation of a polyunsaturated fragment at m/z 204.1381 ([C_13_H_18_NO]^+^). In addition, a fragment with a m/z of 112.0751 (a ketene) was detected, the formation of which was described in the DP1 amine and followed a similar route.

### Evolution kinetics of DPs

Samples from photodegradation experiments conducted with amended and unamended paddy soil and paddy water were subjected to analysis in order to monitor the evolution of the DPs. All of the by-products were observed to possess a greater polarity than the parent compound. We observed the same byproducts and similar evolution trends in all the matrices studied. Figure 3A shows an example of the evolution trends in an experiment in paddy water. The chromatographic method developed allowed for the detection and separation of five different byproducts from photolyzed solutions of profoxydim.

Figure 3A shows two chromatograms at different times during profoxydim degradation in paddy water. The chromatogram at the top corresponds to 30 min of irradiation, in which four byproducts (DP1, DP2a/DP2b, DP4 and DP3) were observed. At the bottom of Fig. 3A, the second chromatogram corresponds to 4 h of irradiation, in which two new minor DPs were detected: DP5a and DP5b.

For the kinetic profile of the different DPs formed, Fig. 1B shows the evolution of DPs versus irradiation time until the complete transformation of profoxydim was achieved. DP1 and DP2 (DP2a and DP2b) were the main byproducts, and the other DPs that formed in smaller amounts were considered minor products.

The concentration of the main byproduct, DP1 [t_R_ = 27.8 min], increased during the five hours of irradiation; it was the major compound at the end of the experiment and was more stable than the parent. The concentration of DP2 (t_R_ = 23.1 min and t_R_ = 38.2 min) increased rapidly to a maximum during the first hour of irradiation and thereafter decreased slowly and remained stable for the remainder of the experiment. These byproducts were very stable and have the potential to accumulate in the environment. Thus, these data highlight the importance of conducting further studies on these DPs of profoxydim. DP3 [t_R_ = 44.2 min], DP4 [t_R_ = 31.6 min] and DP5 [t_R_ = 5.8 min t_R_ = 6.9 min] were minor compounds.

In comparison to the biological degradation of profoxydim in soil in the absence of light, the profile and evolution of common DPs exhibited distinct characteristics^7^. Profoxydim-sulfoxide (DP2) was identified as the predominant and most stable compound generated by microbial processes, whereas profoxydim-imine (DP1), the primary compound generated by photodegradation, was not detected, suggesting that the oxime group was cleaved by solar radiation.

### Mechanisms and photodegradation pathway

**Fig. 5:**
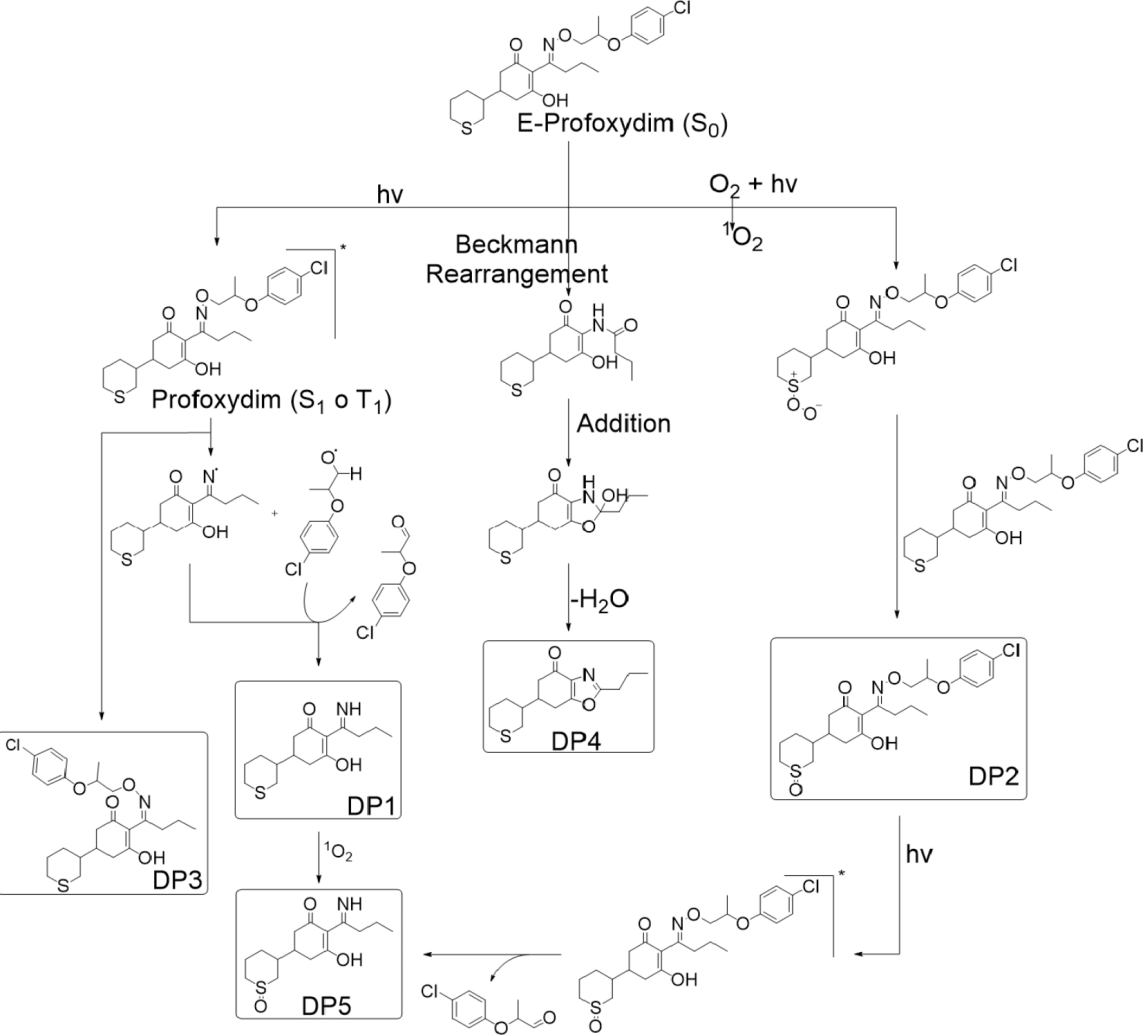
Proposed photodegradation pathway of profoxydim.

The formation mechanisms of herbicide degradation products (DPs) are often complex, depending on both the functional groups present and the molecular structure of the herbicide. As illustrated in Fig. 5, the photodegradation of profoxydim in a paddy system leads to the formation of various compounds, which can be categorized into three groups based on their formation processes: oxime E/Z photoisomerization, N–O bond photolysis, and oxidative photoinduced reactions. Although most degradation products of profoxydim are photodegradation products, a small amount of the oxazole DP4 was detected, which is typically described in the literature as a thermal degradation product.

Upon exposure to visible light, profoxydim transitions from its ground state to an excited singlet (S1) or triplet (T1) state, making the molecule more prone to transformation. The excited form of E-profoxydim undergoes photoisomerization, yielding Z-profoxydim (DP3). A dynamic equilibrium exists between E- and Z-profoxydim, as previously described by our research group for photodegradation processes involving cyclohexanediones such as alloxydim^10^ and clethodim^9^. Computational optimization of each tautomer’s conformational landscape using CENSO and ORCA programs^50,51^ with the def2-mSVP basis set, an efficient method for geometry optimization, revealed that the E isomer predominates in all media over the Z isomer. In aqueous environments, the E isomer is the most stable, while the Z isomer is approximately 0.8 kcal/mol higher in Gibbs free energy, maintaining continuous equilibrium between the two forms and complicating their separation.

The excited profoxydim can also degrade via homolytic cleavage of the N–O bond, generating two radicals. The imine DP1 can form when the corresponding ·OR radical abstracts a hydrogen, releasing an aldehyde. Alternatively, hydrogen abstraction from a water molecule in the reaction medium releases a hydroxyl radical (·OH). This type of N-O bond cleavage has been reported for pesticides with similar structures^47,52,53^.

Photoinduced oxidation processes have also been documented for various cyclohexanedione herbicides^49^. These reactions commonly involve the oxidation of sulfides to sulfoxides, initiated by the photoactivation of triplet oxygen to singlet oxygen^54^. The reactive singlet oxygen can oxidize sulfide to peroxysulfoxide, which further reacts with profoxydim, forming the sulfoxide DP2^55^. Additionally, oxidation occurs on profoxydim and the imine DP1, resulting in the formation of DP5. The DP5 decomposition product arises from a combination of photolysis and photoinduced oxidation, though the sequence of these processes remains undetermined.

The oxazole DP4 was detected as a degradation product. The mechanism of its formation, previously described for herbicides with a cyclohexanedione structure such as alloxydim^52^, cycloxydim^48^, and sethoxydim^56^, begins with a Beckmann rearrangement leading to an amide. This amide undergoes nucleophilic attack from the enol oxygen, forming a new ring, and the subsequent elimination of a water molecule results in DP4. In light of the outcomes yielded, it can be proposed that the reactions involved in the photolysis and the degradation route may be suitable or extended to compounds exhibiting a comparable structural and moieties-related profile, including sulphur atoms and oxime groups.

## Conclusions

This work represents a valuable contribution to the study of the environmentally relevant photodegradation of profoxydim under conditions representative of a rice paddy field. The half-life of profoxydim in paddy water and soil was found to be relatively short, indicating that it undergoes rapid photodegradation under simulated sunlight conditions. The addition of BC as an organic amendment has been demonstrated to exert an inhibitory effect on the degradation of the herbicide. The unambiguous characterization of five DPs and their kinetic evolutions provided insights into the pathways of profoxydim photodegradation. These findings emphasise the necessity of closely monitoring the products resulting from the degradation of profoxydim and the importance of conducting a risk assessment to evaluate their potential impacts.

This comprehensive understanding of the persistence of profoxydim in a paddy rice system will facilitate risk assessments and the implementation of management strategies to protect the environment.

## Supplementary Information

Below is the link to the electronic supplementary material.

Supplementary Information.

## Data Availability

Data availabilityAll data generated or analysed during this study are included in this published article and data presented in this study are available on request from the corresponding author (Pilar Sandin-España: sandin@inia.csic.es).
