# Supplementary material for: Kinetic study, byproducts characterization and photodegradation pathway of profoxydim in a biochar water soil system
Source: Sci Rep. 2024 Nov 7;14:27117. doi: 10.1038/s41598-024-78621-x (PMC11543925; doi:10.1038/s41598-024-78621-x)
Supplement: Supplementary file 1 — Supplementary Information. [file 41598_2024_78621_MOESM1_ESM.docx]

| Paddy soil | Seville | Biochar |
| --- | --- | --- |
| pH (suspended 2:5 in water) | 8.15 | 10.33 |
| Organic matter oxidizable (%) | 1.26 | 6.49 |
| Clay (%) D < 0.002 mm | 30.8 | - |
| Silt (%) 0.002 < D < 0.05 mm | 14.0 | - |
| Sand (%) 0.05 < 2 mm | 55.2 | - |
| N_total_ (%) | 0.10 | 0.67 |
| P (mg kg^-1^) | 1.02 | 128 |
| K (mg kg^-1^) | 253 | 7730 |
| Mg (mg kg^-1^) | 225.5 | 874 |
| Na (mg kg^-1^) | 85.9 | 292 |

**Table S1.1.** Physicochemical characterization of paddy soil used in this study.

| Parameters | Ultrapure water | Paddy water | Paddy water amended BC |
| --- | --- | --- | --- |
| pH | 7.6 ± 0.2 | 8.5 ± 0.2 | 8.7 ± 0.2 |
| Cloruros | 0.160 mg/L | 221 mg/L | 177 mg/L |
| Sulfatos | < 0.10 mg/L | 56.1 mg/L | 61.4 mg/L |
| Nitratos | < 0.10 mg/L | < 0.10 mg/L | < 0.10 mg/L |
| Nitritos | < 0.10 mg/L | < 0.10 mg/L | < 0.10 mg/L |
| Fosfatos | 0.700 mg/L | < 0.15 mg/L | < 0.15 mg/L |
| Sólidos en suspensión | < 50.0 mg/L | 68.8 ± 3.9 mg/L | 65.4 ± 3.7 mg/L |
| Carbono orgánico total | 1.50 mg/L | 71.0 mg/L | 102.4 mg/L |
| Carbonatos | < 5 mg/L CaCO_3_ | 50.0 mg/L CaCO_3_ | 50.0 mg/L CaCO_3_ |
| Bicarbonatos | < 6 mg/L HCO_3_ | 116 mg/L HCO_3_ | 12.2 mg/L HCO_3_ |
| Sólidos totales disueltos | 1.84 mg/L | 609 mg/L | 503 mg/L |
| Salinidad | < 0.0002 ‰ | 0.399 ‰ | 0.320 ‰ |
| Calcio | < 0.500 mg/L | 169 ± 16 mg/L | 82.9 ± 8.9 mg/L |
| Cobre | < 0.050 mg/L | < 0.050 mg/L | < 0.050 mg/L |
| Hierro | < 5.00 mg/L | 90 ± 14 µg/L | 23.9 ± 3.8 µg/L |
| Magnesio | < 0.500 mg/L | 20.5 ± 2.4 mg/L | 17.7 ± 2.1 mg/L |
| Sodio | < 0.500 mg/L | 131 ± 13 mg/L | 111 ± 12 mg/L |

**Table S1.2.** Physicochemical characterization of paddy waters used in this study.


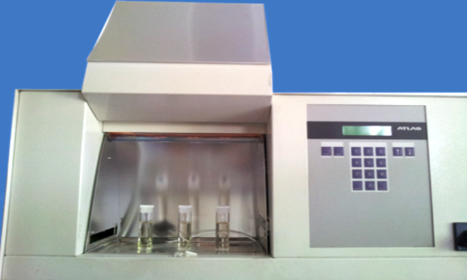

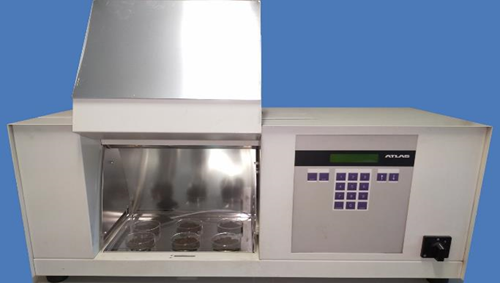


**Fig. S1.** Experimental device of photodegradation in water (a) and soil (b) samples.


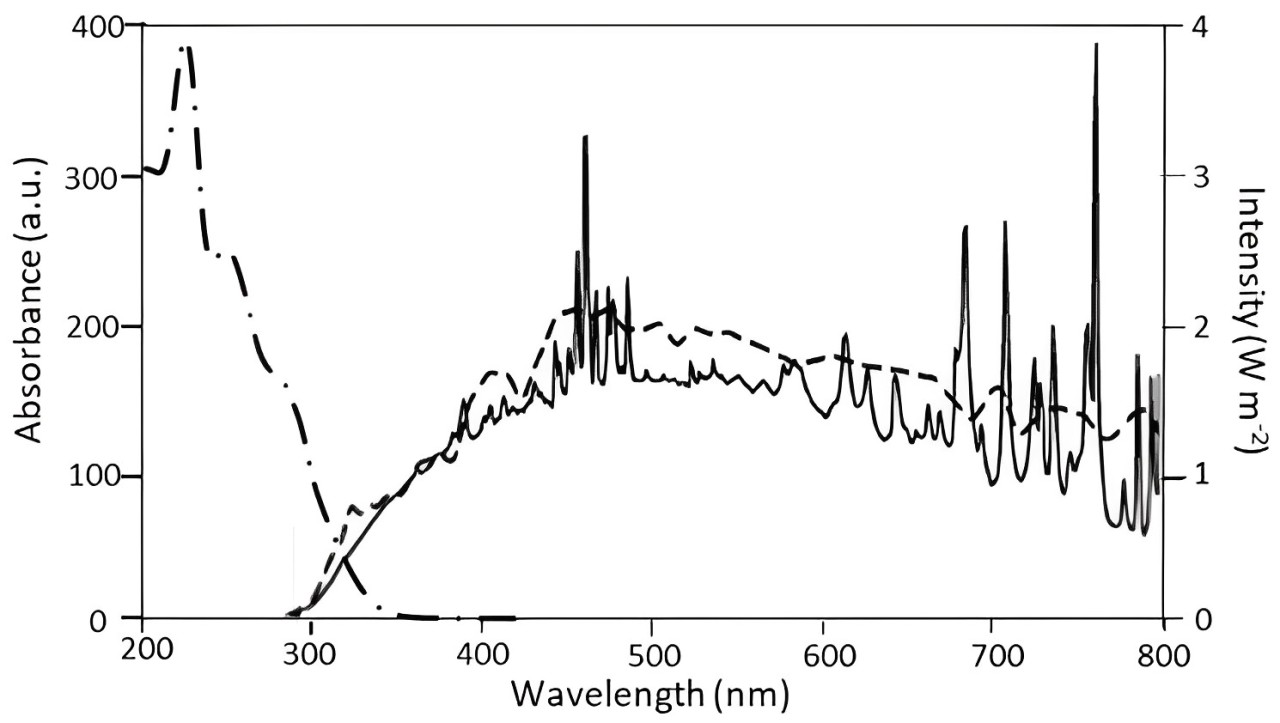


**Fig. S2.** UV spectra of profoxydim (**— · — · —**), Xenon lamp simulated light emission (**— —**) and typical solar irradiance (**——**).


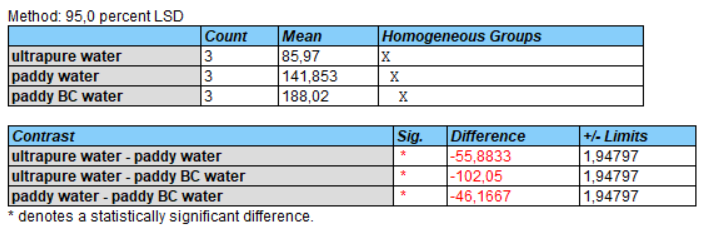

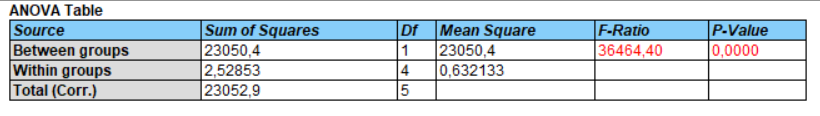

**Fig S3.** ANOVA´s results of water samples in photodegradation of profoxydim.


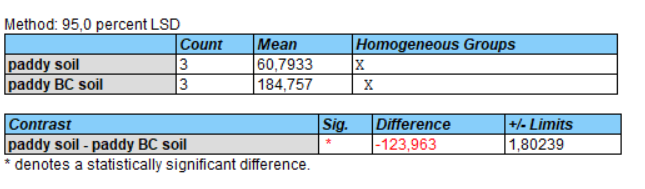

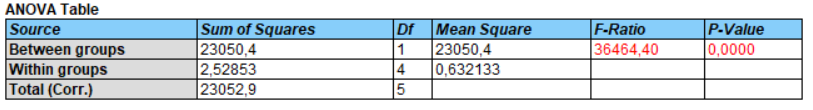

**Fig 4.** ANOVA´s results of soil samples in photodegradation of profoxydim.

**Fig. S5.** Residuals results of the different fittings of profoxydim degradation in all photodegradation experiments (water samples: a)ultrapure, b)paddy and c)paddy water amended with BC; soil samples: d)paddy soil and e)amended paddy soil with BC).
